# Effects of the source of information and knowledge of dengue fever on the mosquito control behavior of residents of border areas of Yunnan, China

**DOI:** 10.1186/s13071-023-05916-9

**Published:** 2023-09-01

**Authors:** Xinchang Lun, Rui Yang, Linghong Lin, Yiguan Wang, Jun Wang, Yuhong Guo, Pengcheng Xiu, Caiying Zhu, Qiyong Liu, Lei Xu, Fengxia Meng

**Affiliations:** 1 National Key Laboratory of Intelligent Tracking and Forecasting for Infectious Diseases, National Institute for Communicable Disease Control and Prevention, Chinese Center for Disease Control and Prevention, Beijing, 102206 People’s Republic of China; 2 Yunnan Institute of Parasitic Diseases Control, Pu´er, 665000 Yunnan People’s Republic of China; 3 Fuzhou Center for Disease Control and Prevention, Fuzhou, 350004 Fujian People’s Republic of China; 4 Changsha Center for Disease Control and Prevention, Changsha, 410004 Hunan People’s Republic of China; 5 Vanke School of Public Health, Tsinghua University, Beijing, 100084 People’s Republic of China

**Keywords:** Information sources, Dengue fever knowledge, Mosquito control behavior, Influential factors

## Abstract

**Background:**

Strengthening the mosquito control measures undertaken by residents of an area where dengue fever is present can significantly decrease the spread of this disease. The aim of this study was to explore the effects of the source of information and knowledge of dengue fever on the mosquito control behavior of residents of areas at high risk of this disease to determine effective ways of enhancing this behavior.

**Methods:**

A survey was conducted via face-to-face interviews or questionnaires between March and May 2021 in three regions of the province of Yunnan, China. The survey included basic information about the respondents, the source(s) of their dengue fever information, the level of their dengue fever knowledge, and the measures they had implemented to control mosquitoes. Principal component analysis was used to extract the main components of the sources of information. Correlation analysis and structural equation analysis were used to explore the impact of the sources of information and residents’ dengue fever knowledge on their mosquito control behavior.

**Results:**

Publicity achieved through mass media, including official WeChat accounts, magazines/newspapers, poster leaflets, television/radio and the Internet, had a direct effect on dengue fever knowledge and mosquito control behavior, and indirectly affected mosquito control behavior through dengue fever knowledge. Organized publicity campaigns, including information provided by medical staff and through community publicity, had a direct effect on dengue fever knowledge and indirectly affected mosquito control behavior through dengue fever knowledge. The residents’ level of dengue fever knowledge had a significant, positive, direct effect on their mosquito control behavior.

**Conclusions:**

Mosquito control is an important measure for the prevention and control of outbreaks of dengue fever. An effective source of information can improve the level of dengue fever knowledge among residents and thus enhance their mosquito control behavior.

**Graphical Abstract:**

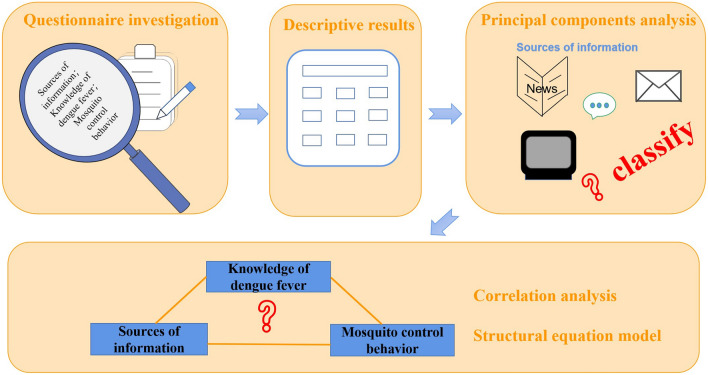

**Supplementary Information:**

The online version contains supplementary material available at 10.1186/s13071-023-05916-9.

## Background

Dengue fever is caused by infection with the dengue virus, which is transmitted through the bite of a female *Aedes* mosquito [1, 2]. Dengue fever is characterized by fever, headache, myalgia, arthralgia, rash, and other clinical symptoms and signs. It can develop into potentially fatal severe dengue hemorrhagic fever, which is also known as dengue shock syndrome, with bleeding, thrombocytopenia and plasma leakage [3, 4]. The present dengue fever epidemic has expanded dramatically since 2012 into 128 countries [5]. The total global cost of dengue fever was estimated to be $8.9 billion in 2013 [6], and vector-borne diseases were identified as one of the top 10 threats to global health by the World Health Organization in 2019 [7].

The pathogenesis of dengue fever remains unclear, and there is no specific therapeutic medicine for the disease. The development of a vaccine for dengue has encountered some obstacles [8, 9]. CYD-TDV, the first and, to this day, only dengue fever vaccine, which was licensed in 2015, is highly effective for people with a history of dengue infection, but for seronegative individuals, it unexpectedly increases the risk of developing severe dengue fever after natural infection with the virus [10]. With no effective dengue fever vaccine or antiviral treatment for the general population, the prevention of dengue fever relies primarily on effective measures of vector control [11, 12], which can limit the spread of dengue virus by reducing people’s contact with its vectors [13].

Yunnan lies in the southwestern part of China, bordering Myanmar, Laos, and Vietnam, and connects Southeast Asia with South Asia, where dengue fever is endemic [14]. Cases of dengue fever in China are more likely to be imported. During 2005 and 2019, Yunnan experienced a considerable number of imported dengue fever cases, and the southwestern border area of the province was identified as the hotspot [15]. This area experiences high temperatures and levels of precipitation throughout the year, which facilitate the reproduction of *Aedes* mosquitoes. Once dengue fever has been imported into an area, it can easily lead to local transmission and an epidemic in the presence of a sufficient number of mosquito vectors.

Implementing vector control measures at the household level can reduce the risk of dengue infection, and all members of a household should be encouraged to practice these measures [16]. Measures designed to prevent contact between humans and vectors of dengue include the use of household insecticides (e.g., insecticide coils, mats and liquids against mosquitoes) and mosquito nets, installation of door and window screening, use of mosquito repellents, the wearing long-sleeved clothing and trousers, changing water storage behavior, and removing potential vector-breeding containers, in addition to other measures that target immature and adult vectors. The willingness of locals to implement these vector-preventive behaviors positively correlates with dengue fever knowledge, as reported from Guyana [17] and Cuba [18]. A survey of 280 residents in an outbreak area of dengue fever in Malaysia showed that sources of information, mass media, local contacts and sessions where residents were directly provided with information, played a key role in providing people with knowledge of dengue fever [19]. Another dengue fever survey conducted in four villages in Kuala Kangsar, Malaysia, found that respondents who learned about dengue fever through television or radio had a higher level of knowledge about this disease and *Aedes* mosquitoes [20]. Thus, the source of information may significantly impact a persons’s understanding of dengue fever and the measures that they take for mosquito avoidance.

Understanding the relationships between the source of information, the level of dengue fever knowledge and the level of mosquito control behavior can guide policy makers so that they can obtain funds to improve public awareness of how to control mosquitoes and thus prevent dengue fever outbreaks. Mosquito control behavior refers to the use of insecticides, screens and other means to control biting mosquitoes. Via face-to-face interviews and questionnaires, we carried out a survey of 721 residents in Yunnan regarding their knowledge of dengue fever and mosquitoes. Using Spearman correlation and structural equation analysis, we studied the influence of sources of information and dengue fever knowledge on residents’ mosquito control behavior.

## Methods

### Data collection

The data were collected from March to May 2021. Staff from the local Centers for Disease Control and Prevention were given professional training before conducting the survey, including familiarity with the questionnaire contents, knowledge of survey skills, and information for standardization of the questionnaire’s completion. Respondents who could read the questionnaire completed the questionnaire by themselves. For the respondents who were unable to read the questionnaire, a member of survey staff read the questionnaire out to them and helped them to record their answers.

**Table 1:** Questionnaire contents

Sources of information	Knowledge of dengue fever	Mosquito control behavior
(1) Close family members (2) Other relatives, and friends (3) Community publicity (4) Medical staff (5) Television or radio (6) Magazines or newspapers (7) Poster leaflets (8) The Internet (9) Official WeChat accounts	(1) Dengue fever is transmitted by mosquitoes (2) Fever is a symptom of dengue fever (3) Headache is a symptom of dengue fever (4) Arthralgia is a symptom of dengue fever (5) Rash is a symptom of dengue fever (6) Bleeding is a symptom of dengue fever (7) Fatigue is a symptom of dengue fever (8) Mosquito larvae live in water	(1) Use of mosquito repellent (2) Use of mosquito nets (3) Screening for doors and windows (4) Use of insecticides (5) Use of mosquito coils (6) Wearing long-sleeved clothing and trousers (7) Removing indoor and outdoor garbage and water (8) Removing shrubs and weeds from around the place of residence (9) Water storage

Three border regions in Yunnan were selected for the survey based on the epidemiological characteristics of cases of dengue fever in them, as follows: Simao, which has had a few imported cases and a few local outbreaks; Ruili, which has had many imported cases but few local outbreaks; and Jinghong, which has had few imported cases but many local outbreaks. Field surveys were conducted in areas with significant population mobility, which gave the additional advantages of crowd representativeness, convenience for carrying out a survey, a high risk of dengue fever, and wide crowd coverage (specific data collection areas are given in Additional file 1).

### Included variables

The first draft of the questionnaire was designed after reviewing the relevant literature and taking into consideration the purpose of the survey. Then, experts from relevant fields who had participated in, and were familiar with, dengue fever prevention and control were invited to modify and finalize the questionnaire, the contents of which are shown in Table 1.

### Statistical analyses

Principal component (PC) analysis (PCA) was used to extract the main components of the sources of information. PCA is used to reduce the dimensionality of data, and is often used to analyze the correlation between each item and variable to determine the PCs with high correlations [21]. Using the PCA method, nine sources of information were summarized and dimensionally reduced, and several representative comprehensive variables were extracted. The extracted components were independent of each other. Spearman correlation coefficient was used to analyze the correlations between residents’ dengue fever knowledge and mosquito control behavior and sources of information, and to explore the information source factors that influenced residents’ dengue fever knowledge and level of mosquito control behavior. Finally, a structural equation model (SEM) was used to analyze the influence of sources of information and residents’ dengue fever knowledge on their mosquito control behavior. A SEM is a statistical analysis method based on the covariance matrix of variables used to analyze the relationship between variables, and combines two statistical techniques: factor analysis and path analysis. These analyses can show the relationship between latent variables (unobservable variables or theoretical variables) and between latent variables and observed variables [22]. Two models were used: a structural model and a measurement model [23]. The structural model was used to evaluate the relationship between latent variables, while the measurement model was used to describe the relationship between observed variables and the measurement model. SEM has many advantages; for example, it can simultaneously process latent variables (variables that cannot be directly measured) and observable variables (variables that can be directly measured), simultaneously consider and process multiple dependent variables, allow measurement errors between dependent variables and independent variables, estimate factor structure and factor relationship at the same time, and can be used to compare the goodness of fit of different models [24]. The χ^2^/*df* ratio, root mean square error of approximation (RMSEA), goodness-of-fit index (GFI), adjusted goodness-of-fit index (AGFI), comparative fit index (CFI) and incremental fit index (IFI) were used to evaluate the goodness of fit of the model [25–27]. We evaluated goodness of fit by using the following criteria: χ^2^/*df* < 3, RMSEA < 0.08, GFI > 0.90, AGFI > 0.90, CFI > 0.90, and IFI > 0.90 [28–30].

All statistical analyses were conducted using R v.4.0.5 software.

### Hypotheses

We proposed the following primary hypotheses: (i) the information source is directly associated with residents’ mosquito control behavior, and (ii) residents’ dengue fever knowledge is directly associated with their mosquito control behavior.

## Results

### Descriptive results

#### Basic information

Basic information about the respondents is presented in Table 2. We collected this information from the 721 respondents in the survey. More than half of the respondents (60.06%, 433/721) were women. The majority of respondents (56.45%, 407/721) were aged between 31 and 50 years. In total, 52.15% (376/721) of the respondents were business service providers. In terms of education, the proportion of respondents (31.21%, 225/721) with a junior high school education was the highest.

**Table 2:** Basic information about the respondents

Variables	Total no. (%) (*n* = 721)	No. in Simao (%) (*n* = 153)	No. in Ruili and Jinghong (%) (*n* = 568)	*P*-value
Gender				
Male	288 (39.94%)	67 (43.79%)	221 (38.91%)	0.274
Female	433 (60.06%)	86 (56.21%)	347 (61.09%)	
Age				
11–30 Years old	222 (30.79%)	73 (47.71%)	149 (26.23%)	< 0.001
31–50 Years old	407 (56.45%)	74 (48.37%)	333 (58.63%)	
51 Years old and above	92 (12.76%)	6 (3.92%)	86 (15.14%)	
Occupation				
Business services	376 (52.15%)	92 (60.13%)	284 (50.00%)	0.026
Other	345 (47.85%)	61(39.87%)	284 (50.00%)	
Education				
Primary school and below	120 (16.64%)	3(1.96%)	117 (20.60%)	< 0.001
Junior high school	225 (31.21%)	29 (18.95%)	196 (34.51%)	
Senior school	175 (24.27%)	36 (23.53%)	139 (24.47%)	
University and above	201 (27.88%)	85 (55.56%)	116 (20.42%)	

#### Sources of information

Among the nine sources of information, the number of people who learned about dengue fever through community publicity was the highest (59.78%, 431/721), followed by relatives/friends (30.37%, 219/721) and the Internet (27.88%, 201/721; Table 3). Community publicity is a common means of disseminating knowledge on the prevention and control of dengue fever among residents by community workers, including regular visits to residents to disseminate basic information about dengue fever, demonstrate dengue fever control practices, discover problems that arise in their practice, and evaluate control effectiveness. Official WeChat accounts and magazines/newspapers were less frequently used to obtain information on dengue fever, and accounted for 8.88% (64/721) and 8.74% (63/721) of the sources used by the surveyed residents, respectively.

**Table 3:** Sources of information for knowledge on dengue fever

Variables	Total no. (%) (*n* = 721)	No. in Simao (%) (*n* = 153)	No. in Ruili and Jinghong (%) (*n* = 568)	*P*-value
Through close family	144 (19.97%)	17 (11.11%)	127 (22.36%)	0.002
Through other relatives, or friends	219 (30.37%)	43 (28.10%)	176 (30.99%)	0.492
Through community publicity	431 (59.78%)	60 (39.22%)	371 (65.32%)	< 0.001
Explained by medical staff	196 (27.18%)	41 (26.80%)	155 (27.29%)	0.903
Through television and radio	193 (26.77%)	39 (25.49%)	154 (27.11%)	0.687
Through magazines and newspapers	63 (8.74%)	23 (15.03%)	40 (7.04%)	0.002
Through poster leaflets	113 (15.67%)	16 (10.46%)	97 (17.08%)	0.046
Through the Internet	201 (27.88%)	53 (34.64%)	148 (26.06%)	0.036
Through official WeChat accounts	64 (8.88%)	10 (6.54%)	54 (9.51%)	0.251

#### Knowledge of dengue fever

Up to 93.62% (675/721) of the residents knew that dengue fever was transmitted by mosquitoes, and 90.85% (655/721) knew that fever was a symptom of dengue fever (Table 4). A large proportion of residents (77.39%, 558/721) knew that mosquito larvae lived in water. However, the surveyed residents did not fully realize there were other dengue fever symptoms apart from fever. For example, only 16.37% (118/721) and 8.74% (63/721) recognized rash and bleeding as dengue fever symptoms, respectively.

**Table 4:** Knowledge of dengue fever among residents

Variables	Total no. (%) (*n* = 721)	No. in Simao (%) (*n* = 153)	No. in Ruili and Jinghong (%) ( *n* = 568)	*P*-value
Dengue fever is transmitted by mosquitoes	675 (93.62%)	130 (84.97%)	545 (95.95%)	< 0.001
Fever is a symptom of dengue fever	655 (90.85%)	136 (88.89%)	519 (91.37%)	0.344
Headache is a symptom of dengue fever	386 (53.54%)	76 (49.67%)	310 (54.58%)	0.280
Arthralgia is a symptom of dengue fever	338 (46.88%)	52 (33.99%)	286 (50.35%)	< 0.001
Rash is a symptom of dengue fever	118 (16.37%)	24 (15.69%)	94 (16.55%)	0.798
Bleeding is a symptom of dengue fever	63 (8.74%)	17 (11.11%)	46 (8.10%)	0.242
Fatigue is a symptom of dengue fever	215 (29.82%)	34 (22.22%)	181 (31.87%)	0.021
Mosquito larvae live in water	558 (77.39%)	86 (56.21%)	472 (83.10%)	< 0.001

#### Mosquito control behavior

A large number of residents had used mosquito coils, mosquito repellents, and mosquito nets to prevent mosquito bites, accounting for 69.35% (500/721), 68.38% (493/721) and 52.29% (377/721) of residents, respectively (Table 5). However, only one-fifth (151/721) of residents resorted to removing shrubs, weeds and water containers from around their settlements as measures to prevent mosquitoes from breeding.

**Table 5:** Mosquito control behavior among residents

Variables	Total no. (%) (*n* = 721)	No. in Simao (%) (*n* = 153)	No. in Ruili and Jinghong (%) (*n* = 568)	*P*-value
Use of mosquito repellent	493 (68.38%)	105 (68.63%)	388 (68.31%)	0.94
Use of mosquito nets	377 (52.29%)	79 (51.63%)	298 (52.46%)	0.855
Use of screening for doors and windows	296 (41.05%)	65 (42.48%)	231 (40.67%)	0.685
Spraying insecticides	230 (31.90%)	25 (16.34%)	205 (36.09%)	< 0.001
Use of a mosquito coil	500 (69.35%)	81 (52.94%)	419 (73.77%)	< 0.001
Wearing long-sleeved clothing and trousers	178 (24.69%)	36 (23.53%)	142 (25.00%)	0.708
Removing indoor and outdoor garbage and water	338 (46.88%)	61 (39.87%)	277 (48.77%)	0.05
Removing shrubs and weeds from around the place of residence	151 (20.94%)	20 (13.07%)	131 (23.06%)	0.007
Not storing water	138 (19.14%)	10 (6.54%)	128 (22.54%)	< 0.001

### Principal components analysis

Three PCs were extracted from the sources of information based on the eigenvalues (> 1), and represented 52% of the original variation (Table 6). Nine sources of information were summarized according to the loading of each for the three PCs. PC1 was associated with obtaining information through official WeChat accounts, magazines and newspapers, poster leaflets, television/radio, and the Internet. We named this component “mass media publicity.” PC2 was associated with obtaining information through relatives/friends and close family. We named this component “familiar person publicity.” PC3 was associated with obtaining information through explanations given by medical staff and through community publicity. We named this component “organized publicity.”

**Table 6:** Contribution of the various sources of information to the principal components

	Component		
	1	2	3
Official WeChat accounts	0.74	0.04	− 0.04
Magazines and newspapers	0.64	0.03	− 0.10
Poster leaflets	0.64	− 0.02	− 0.22
Television and radio	0.61	− 0.15	0.03
The Internet	0.50	0.12	− 0.48
Other relatives, or friends	0.08	0.77	0.20
Close family	0.17	0.70	0.32
Explained by medical staff	0.40	− 0.11	0.60
Community publicity	0.27	− 0.48	0.52

### Correlation analysis

Spearman correlation was used to analyze the relationship between dengue fever knowledge, mosquito control behavior and three PCs: mass media publicity, familiar person publicity and organized publicity. Dengue fever knowledge was significantly correlated with mass media publicity and organized publicity (Table 7), which suggested that residents’ dengue fever knowledge benefited from these types of publicity. Meanwhile, mosquito control behavior also showed significant positive correlations with these two PCs. In contrast, familiar person publicity did not correlate with dengue fever knowledge and mosquito control behavior, suggesting that information from relatives/friends or close family contributed little to people’s understanding of dengue fever and mosquitoes.

**Table 7:** Correlation matrix between dengue fever knowledge, mosquito control behavior and sources of information

Variables	Mass media publicity	Familiar person publicity	Organized publicity
Dengue fever knowledge	0.304**	− 0.034	0.144**
Mosquito control behavior	0.390**	− 0.032	0.113**

**P < 0.01

### Structural equation model

Table 8 shows the overall fit of the model, which indicated that the data were adequately fitted according to the following criteria (see “Methods”): χ^2^/*df* < 3, RMSEA < 0.08, GFI > 0.90, AGFI > 0.90, IFI > 0.90, and CFI > 0.90.

**Table 8:** Goodness-of-fit indices of the structural equation model (SEM)

Model	*χ*^2^/*df*	RMSEA	GFI	AGFI	IFI	CFI
Mass media publicity	2.241	0.041	0.952	0.938	0.913	0.912
Familiar person publicity	2.220	0.041	0.952	0.938	0.907	0.906
Organized publicity	2.321	0.043	0.950	0.936	0.901	0.900

Table 9 shows the effect of three sources of information on residents’ dengue fever knowledge and mosquito control behavior. Mass media publicity had a direct positive effect on dengue fever knowledge and mosquito control behavior, and an indirect effect on mosquito control behavior through dengue fever knowledge. Organized publicity had a direct positive effect on dengue fever knowledge and an indirect effect on mosquito control behavior through dengue fever knowledge. However, familiar person publicity did not have an effect on dengue fever knowledge or mosquito control behavior. Dengue fever knowledge had a direct positive effect on mosquito control behavior. These results suggested that the level of dengue fever knowledge and mosquito control behavior of residents could be improved by strengthening mass media publicity and organized publicity.

**Table 9:** Direct, indirect and total effects of the variables on knowledge of dengue fever and mosquito control behavior

	Variables	Dengue fever knowledge			Mosquito control behavior		
		Direct	Indirect	Total	Direct	Indirect	Total
MMP	MMP	0.420***	–	0.420***	0.209***	0.234***	0.443***
	DFK	–	–	–	0.558**	–	0.558**
FPP	FPP	− 0.071	–	− 0.071	− 0.016	− 0.046	− 0.062
	DFK	–	–	–	0.645**	–	0.645**
OP	OP	0.165**	–	0.165**	0.004	0.106***	0.110*
	DFK	–	–	–	0.646**	–	0.646**

*MMP* Mass media publicity, *FPP* familiar person publicity, *OP* organized publicity

*P < 0.05, **P < 0.01, ***P < 0.001

**Fig. 1:**
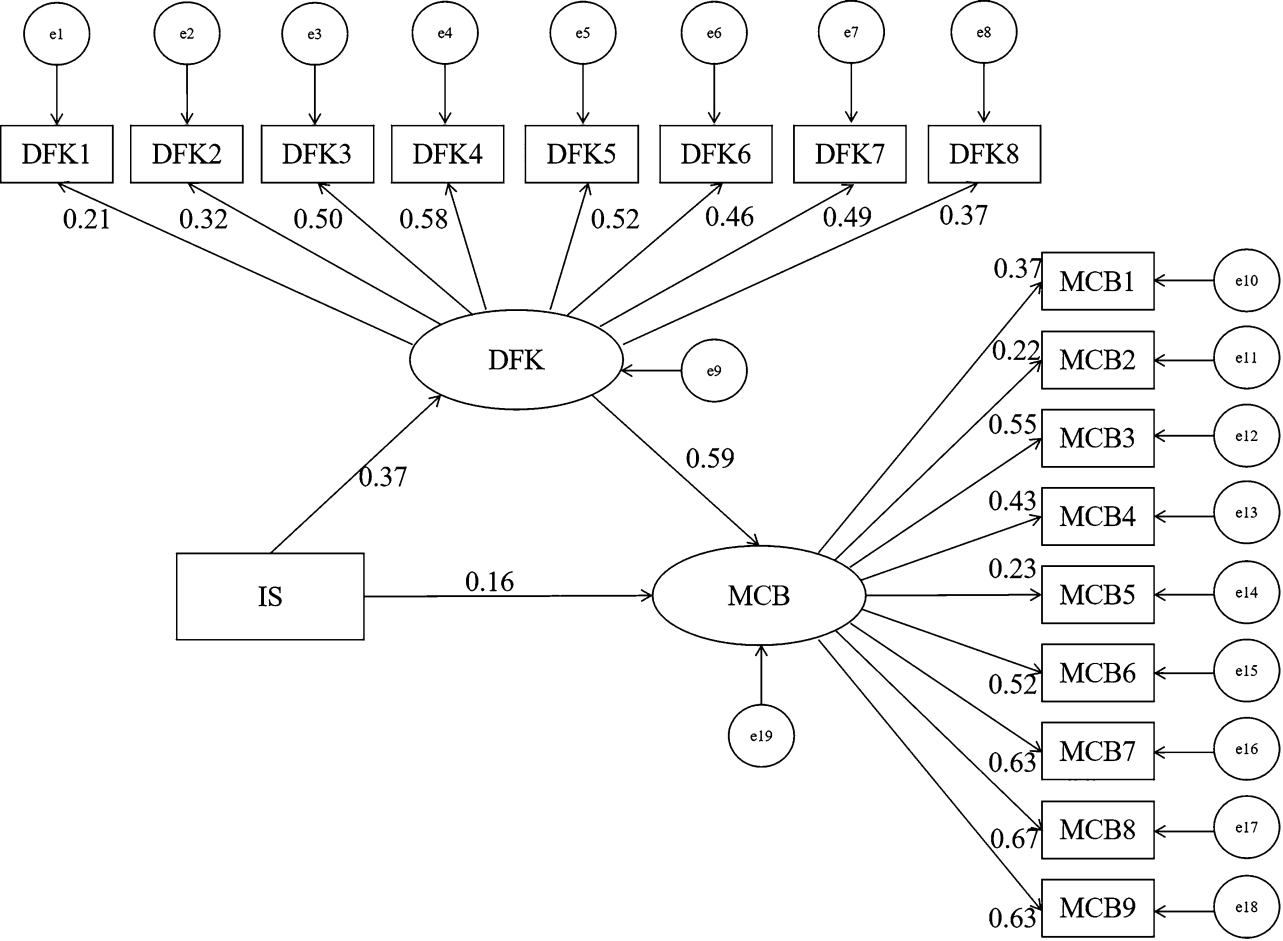
Path diagram of the structural equation model. *IS* Information source, *DFK* dengue fever knowledge, *MCB* mosquito control behavior, *DFK1* dengue fever is transmitted by mosquitoes, *DFK2* fever is a symptom of dengue fever, *DFK3* headache is a symptom of dengue fever, *DFK4* arthralgia is a symptom of dengue fever, *DFK5* rash is a symptom of dengue fever, *DFK6* bleeding is a symptom of dengue fever, *DFK7* fatigue is a symptom of dengue fever, *DFK8* mosquito larvae live in water, *MCB1* use of mosquito repellent, *MCB2* use of mosquito nets, *MCB3* use of screening for doors and windows, *MCB4* use of insecticidal spray, *MCB5* use of mosquito coil, *MCB6* wearing long-sleeved clothing and trousers, *MCB7* removal of indoor and outdoor garbage and water, *MCB8* removal of shrubs and weeds from around the place of residence, *MCB9* no storage of water

Figure 1 shows the path coefficients of the SEM. All sources of information taken together was positively correlated with dengue fever knowledge (β = 0.37, *P* < 0.001) and mosquito control behavior (β = 0.16, *P* < 0.01). These results suggested that all of the sources of information taken together could contribute to a higher level of dengue fever knowledge and mosquito control behavior. Dengue fever knowledge was also positively correlated with mosquito control behavior (β = 0.59, *P* < 0.001), suggesting that a higher level of dengue fever knowledge may enhance mosquito control behavior.

Table 10 shows information on the overall goodness-of fit of the SEM. The indices showed that the data adequately fit the model based on the model testing standards used here.

**Table 10:** Goodness-of-fit indices of the SEM

Goodness of fit	*χ*^2^/*df*	RMSEA	GFI	AGFI	IFI	CFI
Value	2.267	0.042	0.952	0.938	0.909	0.908

Table 11 shows the effect of all of the sources of information taken together on residents’ dengue fever knowledge and mosquito control behavior. The sources of information taken together had a direct positive effect on dengue fever knowledge and mosquito control behavior and an indirect effect on mosquito control behavior through dengue fever knowledge. Meanwhile, dengue fever knowledge had a direct positive effect on mosquito control behavior. These results suggested that increasing the sources of media for the dissemination of information could benefit residents with respect to their knowledge of dengue fever and useful types of behavior for the control of mosquitoes.

**Table 11:** The direct, indirect and total effects of the assessed variables on knowledge of dengue fever and mosquito control behaviors

Variables	Dengue fever knowledge			Mosquito control behavior		
	Direct	Indirect	Total	Direct	Indirect	Total
Information sources	0.367***	–	0.367***	0.155**	0.217***	0.371***
Dengue fever knowledge	–	–	–	0.590**	–	0.590**

**P < 0.01, ***P < 0.001

## Discussion

Travel across national borders was becoming easier and increasing in frequency before the start of the COVID-19 pandemic, and has continued to increase in ease and frequency at the end of COVID-19. Consequently, dengue fever can more easily spread across certain nations, and has thus become a significant threat in them to public health. Strengthening the surveillance of imported cases of dengue fever is of great importance in controlling the spread of this disease. As dengue fever is a mosquito-borne disease, the control of its vectors, *Aedes aegypti* and *Aedes albopictus*, also plays a vital role in its prevention and control. This study surveyed the sources of information on dengue fever, dengue fever knowledge and mosquito control behavior of residents in three border areas of Yunnan as an integrated analysis of the potential factors influencing the latter to inform dengue fever prevention and control programs.

Although there were multiple channels through which the surveyed residents could learn about dengue fever, nearly 60% relied on community-targeted publicity. This result indicated that residents had greater trust in information aimed at the community and were willing to accept publicity and take part in education activities provided in their community. It also showed that community workers had played an essential role in imparting knowledge on dengue fever, which should thus be highlighted as an efficient means of educating the public about dengue fever.

Most of the residents who participated in the survey knew how dengue fever is transmitted and that mosquito larvae live in water. However, they lacked knowledge about some dengue fever symptoms, such as headache, arthralgia, rash, bleeding and fatigue. Publicity on and education about dengue fever symptoms should be strengthened among residents to improve their awareness of these. This knowledge would also encourage patients with relevant symptoms to seek medical advice in good time, and thus improve the early detection of cases of dengue fever in the local population.

Regarding mosquito control behavior among residents, some had implemented measures to prevent mosquito bites, such as the use of mosquito coils, mosquito repellents and mosquito nets. However, few people realized that water in containers, and shrubs and weeds around the household, could be mosquito habitats. Thus most of the residents rarely removed these potential breeding sites, which may have undermined efforts to control mosquitoes. Publicity on dengue fever should also inform people about methods for the control of mosquitoes, such as identifying and removing potential mosquito breeding sites to disrupt the life cycle of these vectors. The control of mosquito breeding sites is also key to controlling the spread of dengue fever. Increasing the frequency of inspection of containers for water accumulation, and overturning containers that hold water, help to control the occurrence and spread of dengue fever.

The results also indicated the importance of carefully selecting sources of information to improve residents’ dengue fever knowledge and mosquito control behavior. Among the three identified PCs, publicity through mass media had a positive effect on dengue fever knowledge and mosquito control behavior, and indirectly affected mosquito control behavior through dengue fever knowledge. Therefore, publicity through mass media, including that achieved through official WeChat accounts, magazines/newspapers, poster leaflets, television and radio, and the Internet, should be increased to improve awareness of dengue fever and increase mosquito control behavior. Interestingly, information from people familiar to the respondents, including close family members and other relatives and friends, did not significantly affect residents’ dengue fever knowledge or mosquito control behavior. This indicated that there may have been little knowledge on dengue fever and mosquitoes among families or friends, or that they all had a similar level of dengue fever knowledge and thus could not benefit from information transmitted between them. In contrast, organized publicity campaigns which involved community physicians or other health workers had a direct effect on dengue fever knowledge and an indirect effect on mosquito control behavior. This highlights the importance of organizing medical staff and community physicians so that they can disseminate information on dengue fever among local residents. These results also showed that officials played an important role in health education, and that the dissemination of information depended on the guidance and drive of officials.

Overall, all of the sources of information taken together had a direct effect on dengue fever knowledge and mosquito control behavior, and an indirect effect on mosquito control behavior through dengue fever knowledge. This indicates that increasing the sources of information can potentially enrich dengue fever knowledge and strengthen mosquito control behavior among residents. Thus, we suggest that the number of channels used to spread information should be increased so that more people become familiar with the symptoms and signs of dengue fever and methods for mosquito control. For example, short videos (e.g., on TikTok) are gaining popularity in all walks of life, and using these to disseminate knowledge about dengue fever is a promising approach. We should also mention the important role played by officials in publicity and education about dengue fever control. Activities can be carried out through a strategy of “small hands holding large hands” to educate children in schools, and students, so that they can learn relevant knowledge about dengue fever and then spread this to their family members so that an increasing number of residents will have a better understanding of dengue fever and take measures to avoid it. We should actively develop sources of information that can effectively improve residents’ knowledge, and identify other means of increasing the dissemination of knowledge, in addition to designing publicity materials aimed at different groups, and regularly evaluate the effects of different sources of information. The channels through which information is disseminated also needs to be adapted according to the results of evaluations, and publicity through effective channels needs to be increased.

Residents’ dengue fever knowledge also had a direct positive effect on their mosquito control behavior. This is consistent with the results of several other studies on improving knowledge of the disease, which can significantly affect behaviors related to its prevention and control [31–33]. The successful prevention and control of dengue fever should begin with the dissemination of knowledge about it among the public in high-risk areas.

A survey of Dai residents in Yunnan Province showed that, when they realize that they are infected with dengue fever, they are willing to first seek treatment from public health institutions [34], which indicates that being able to correctly identify the symptoms of dengue fever has a profound effect on patients’ receiving timely medical treatment. Similarly, having a good understanding of dengue fever and its vector mosquitoes is very important with respect to residents taking appropriate mosquito control measures. Understanding the serious harm caused by infection with the dengue fever virus can greatly stimulate residents’ interest in understanding how to prevent and control dengue fever, which also encourages them to take economical and effective measures to avoid dengue fever infection as far as is possible. Knowledge of the vital role played by mosquitoes in the transmission of dengue fever, and the importance of their breeding grounds, can increase residents’ awareness of how to protect themselves from mosquito bites, as well as lead to improvement in methods used to control mosquitoes.

Yunnan is located in the southwestern border area of China, where there is a high risk of imported cases of dengue fever. In addition, large areas of the region are suitable for the growth and development of mosquitoes due to the hot and rainy climate, which makes this province more prone to local outbreaks of dengue fever. From 2004 to 2018, the average yearly incidence rate of indigenous dengue fever in Yunnan was 0.86 (1/100000), of which indigenous cases in Jinghong and Ruili accounted for 85.1%, and the average yearly incidence rate of imported dengue fever was 0.44 (1/100000) [35]. Thus, this study, which indicates the importance of increasing the sources of information and enhancing dengue fever knowledge for enhanced mosquito control behavior, was carried out in an area where the risk of outbreaks due to local and imported cases is high. We hope that the results of this study will lead to an increase in mass media channels and organized campaigns for the dissemination of information among residents for the prevention and control of dengue fever to reduce outbreaks of this disease.

This study was conducted in border areas with a high risk of imported cases of dengue fever and transmission of the disease. Whether the results of this study are applicable to areas with a low risk of dengue fever needs further exploration. In addition, as this study was a cross-sectional one, causation can only be inferred with caution. Longitudinal studies are necessary, as these may demonstrate a more convincing relationship between dengue fever knowledge and mosquito control behavior.

The findings of this study can be summarized as follows: (i) the source of information, particularly publicity through mass media and organized campaigns, is an important factor with respect to people’s knowledge of dengue fever, and is directly related to their mosquito control behavior; (2) dengue fever knowledge is directly associated with mosquito control behavior, and a higher level of the former can promote the latter.

## Conclusions

Mosquito control is important for the prevention and control of outbreaks of dengue fever. It requires the joint effort of residents, which can be achieved by increasing the number of channels through which they can obtain information on dengue fever, and then by promoting mosquito control behaviors. In particular, the types of mass media for publicity (e.g., official WeChat accounts, magazines/newspapers, poster leaflets, television/radio, and the Internet) should be increased, as should organized publicity campaigns carried out by community physicians and other health workers. We believe that these efforts can help to improve knowledge of dengue fever among residents and promote mosquito control behaviors to effectively reduce the transmission of this disease and thus prevent its spread.

### Supplementary Information

**Additional file 1:** Schematic diagram of survey area.

## Data Availability

The raw data used in this study cannot be shared publicly because it concerns personal information provided by the respondents, who requested that it should be kept private. Readers with any questions on the study can contact the corresponding author.
