# Supplementary figures and images for: Effects of the source of information and knowledge of dengue fever on the mosquito control behavior of residents of border areas of Yunnan, China
Source: Parasit Vectors. 2023 Sep 1;16:311. doi: 10.1186/s13071-023-05916-9 (PMC10472605; doi:10.1186/s13071-023-05916-9)

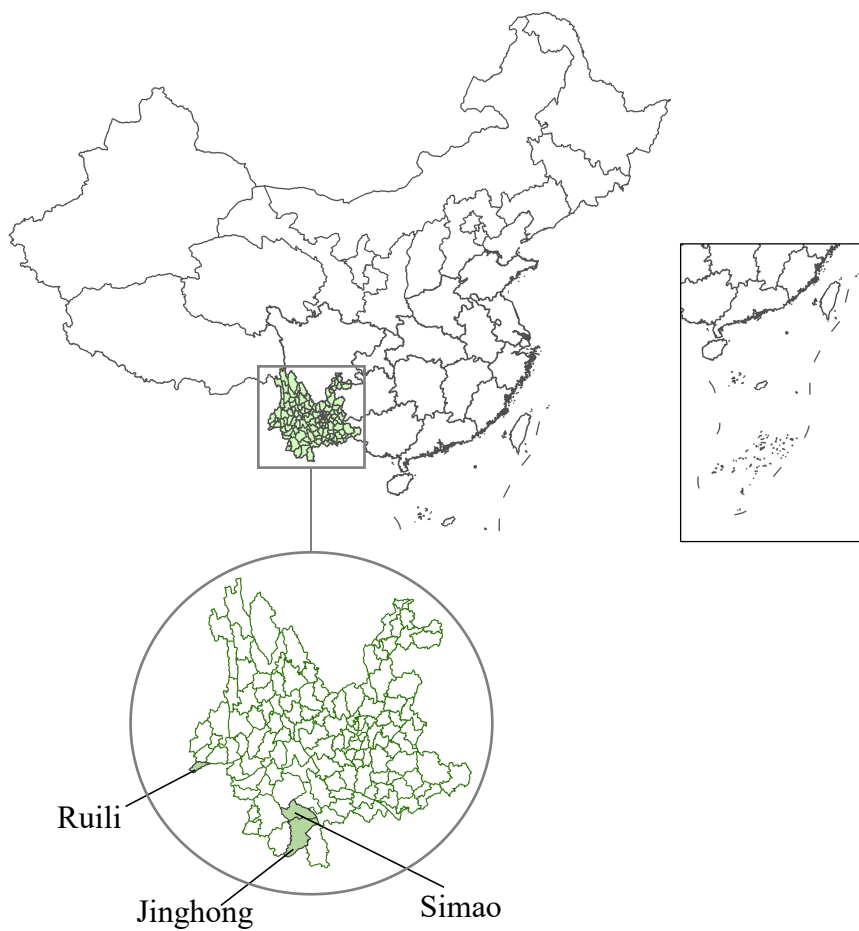

Specific data collection areas

Supplement: Supplementary file 1 — Additional file 1: Schematic diagram of survey area. [file 13071_2023_5916_MOESM1_ESM.pdf]
